# Supplementary material for: Development of Longitudinal, Linked Maternal-Infant Cohorts using the Epic Cosmos Electronic Health Record Dataset
Source: medRxiv. 2026 Jun 4:2026.06.02.26354757. Preprint. [Version 1] doi: 10.64898/2026.06.02.26354757 (PMC13252481; doi:10.64898/2026.06.02.26354757)
Supplement: Supplementary file 1 [file media-1.docx]

**Appendix 1. Steps for creating the birth cohort.**

- Total birth cohort (CY 2023-2024)
  - Using BirthFact table (based on infant records)
- Link to pregnancies
  - Join PregnancyFact (based on maternal records) and BirthFact tables
- Remove historical pregnancies
  - Using PregnancyFact table
- Link to birth encounters during same birth years
  - Join EncounterFact table (2023-2024)
- Remove unavailable birth encounters (i.e., missing, masked, deleted)
  - Using EncounterFact table:
    - if there is a patient (mother) associated with the delivery encounter: patientdurablekey (of mother’s) > 0
    - existence of start date of the encounter (partition key): datekey>0
  - Using Birthfact table:
    - Existence of maternal delivery encounter associated with the birth: motherencounterkey>0
  - Using PregnancyFact table:
    - Existence of the date the clinician created the pregnancy episode (partition key): episodestartdatekey>0
- Identify twins/multiples & assign first infant birth date as the pregnancy delivery date if multiple dates
  - Using BirthFact table: twins/multiples identified as unique IDs with shared PregnancyKey
  - Set the first infant birth date as the delivery date for the shared pregnancy (multiples infant birth dates may differ)
- Exclude any stillbirth pregnancies remaining in error (code present from pregnancy delivery date plus/minus 7 days)
  - ICD-10: O36.4XXO, O36.4XX1, O36.4XX2, O36.4XX3, O36.4XX4, O36.4XX5, O36.4XX9, Z37.1, Z37.3, Z37.4, Z37.6, Z37.7, P95
  - ICD-9: 656.40, 656.41, 656.43, V27.1, V27.3, V27.4, V27.6, V27.7, 768.1
- Assign gestational age (GA)
  - If multiple GAs for a pregnancy in BirthFact table, select lowest
  - If no GA, calculate as delivery date – “pregnancy estimated start date” from the PregnancyFact table
    - The rest are set as missing
- Assign last menstrual period (LMP) (LMP = Delivery date – GA)
  - Rationale is that the delivery date and GA from the BirthFact table are the most reliable

**Appendix 2. Details on variables created.**

Used ICD-10 and ICD-9 diagnosis codes in all diagnosis types (billing, encounter, admitting, discharge diagnosis or problem list) to identify disorders. Codes below indicate “starts with” (e.g., 648.0 includes 648.00-648.04) for brevity.

1. **Maternal characteristics**
   a. Maternal age at delivery: from BirthFact and DurationDim
   b. Racial and ethnic group: from PatientDim, if Hispanic/Latino, classify as such, then classify all without Hispanic/Latino based on racial group. If ethnicity is missing, or not Hispanic/Latino and missing race then classify as missing.
   c. Payer for delivery encounter: from CoverageDim table, ReferenceFinancialClass
   d. Geography: from PatientDim, (1) Northeast; (2) Midwest; (3) South; (4) West; (5) Not U.S. Regions based on US Census Bureau statistical regions (<https://www2.census.gov/geo/pdfs/maps-data/maps/reference/us_regdiv.pdf> )
   e. Rurality: using USDA Rural-Urban Commuting Area Codes and classifications
      i. Metropolitan (1-3), nonmetropolitan (4-10)
   f. Body mass index at delivery: from VitalsFact
      i. Time window: [delivery date -7, delivery date]
      ii. When multiple exact time -> select earliest. If multiple on the soonest exact time choose the min value
      iii. Categorize for descriptive purposes as <30 kg/m^2^; 30 to <40 kg/m^2^; ≥40 kg/m^2^
   g. Parity (prior live births)
      i. (1) nulliparous; (2) 1-4 prior births; (3) 5+ prior births
   h. Chronic hypertension [LMP-90 d, LMP+140 d]
      i. ICD10: I10, O10.0, O11
      ii. ICD9: 401, 642.0, 642.7
   i. Preeclampsia [LMP+140 d, delivery date + 14 d]
      i. ICD10: O11, O14.0, O14.1, O14.2, O15
      ii. ICD9: 642.4, 642.5, 642.6, 642.7
   j. Preexisting diabetes mellitus [LMP, delivery date ]
      i. ICD10: O24.0, O24.1, O24.3, O24.8, Z79.4, E08-E13
      ii. ICD9: 250
   k. Gestational diabetes mellitus [LMP+210 days, delivery date]
      i. ICD10: O24.430, O24.434, O24.435, O24.439
      ii. ICD9: 648.80, 648.81, 648.82, 648.83, 648.84
      iii. Also required no code for preexisting diabetes
   l. Depression [LMP, delivery date ]
      i. ICD10: F32.0-F32.9, F32.A, F33.0-F33.9
      ii. ICD9: 296.2, 296.3, 311
   m. Anemia [delivery date -7 d, delivery date]
      i. Loinc codes: HCT 4544-3 or HGB 718-7
      ii. Hb <11 or Hct<33. Select first if multiple using the exact date if still multiple values on the same time, choose the min value. From labcomponentresultfact
   n. Hemoglobin measurement (mean, SD)
      i. [delivery date -7, delivery date ] - select first if multiple using the exact date if still multiple values on the same time, choose the min value. Join on EncounterKey from labcomponentresultfact. (Hospital Encounters)
   o. Blood pressure measurement during 1^st^ trimester prenatal encounter [LMP, LMP+97 d]
      i. Require during prenatal care encounter as defined elsewhere and select first if multiple using the exact date. If still multiple values, choose the min value.
   p. Blood pressure measurements at delivery admission

[delivery date -7, delivery date ]

i. Select first if multiple using the exact date. If still multiple values,

choose the min value. Join on EncounterKey from VitalFact. (Hospital

Encounters)

2. **Pregnancy & infant characteristics**
   a. Plurality: (1) singleton; (2) twin; (3) triplet or higher order (using PregnancyKey)
   b. Gestational age at delivery: (1) <28 wk; (2) 28 to <32 wk; (3) 32 to <37 wk; (4) 37+ wk
   c. Mode of delivery: (1) spontaneous vaginal; (2) operative vaginal (forceps or vacuum); (3) cesarean
   d. Birthweight: (1) >=4000g; (2) 2500 to <4000 g; (3) 1500 to <2500 g; (4) <1500g
   e. Apgar score at 5 minutes: (1) 0-3; (2) 4-6; (3) 7-10

**Appendix 3. Steps for selection of longitudinal, linked maternal-infant cohorts.**

1. Starting with birth cohort as described in Appendix 1, additionally require ≥1 prenatal care encounter in 1^st^ trimester and ≥1 prenatal care in 2^nd^ trimester
   a. Prenatal care:
      - Encounter type defined as either: “initial prenatal”, “routine prenatal”, or “office visit”
        - For encounters with: Greenlit sources + non-deleted non-masked records + DerivedEncounterStatus: not (“Unspecified”, “Not Applicable”, “Unknown”,” ”,”Invalid”), not deleted from EncounterFact (IsDeleted=0), and has happened at least once (Count=1)
      - In departments either: “Family Medicine”, “Gynecology”, “MFM”, “Midwifery”, “Obstetrics”, “Obstetrics and Gynecology”, “Perinatology”, “Primary Care”
   b. Time windows:
      - 1^st^ trimester: [LMP, LMP+97d]
      - 2^nd^ trimester: [LMP+98, LMP+195d]
2. Additionally require ≥1 postpartum care encounter
   a. Postpartum care:
      - Encounter type defined as either: “Postpartum” or “Office Visit “

- For encounters with: greenlit sources + non-deleted non-masked records + DerivedEncounterStatus should be valid and known
  - In department: “MFM”, “Obstetrics”, “Obstetrics and Gynecology”, “Perinatology”, “Family Medicine”, “Gynecology”, “Midwifery”, “Primary Care”, “Postpartum”
  b. Time window:
     - >delivery date, <delivery date+84d

3. Additionally require ≥1 infant follow-up encounter
a. Infant follow-up:
   - Ensure not a hospital encounter (not ER, not admitted – example to start Encounter type defined as either: “Office Visit”, “Well-Child”
   - In department: “Family Medicine”, “Pediatrics”
b. Time window:
   - >birth hospitalization discharge data, <84d
